# Tocilizumab Exerts Anti-Tumor Effects on Colorectal Carcinoma Cell Xenografts Corresponding to Expression Levels of Interleukin-6 Receptor

**DOI:** 10.3390/ph17010127

**Published:** 2024-01-18

**Authors:** Yuan-Chiang Chung, Szu-Jung Chen, Chiu-Chen Huang, Wei-Chun Liu, Ming-Tsung Lai, Ting-Yu Kao, Wei-Shun Yang, Chien-Hui Yang, Chih-Ping Hsu, Jia-Feng Chang

**Affiliations:** 1 Department of Surgery, Kuang Tien General Hospital, Taichung 433, Taiwan; chung11753@ktgh.com.tw; 2 Department of Surgery, Chung-Kang Branch, Cheng-Ching General Hospital, Taichung 407, Taiwan; 3 Department of Radiation Oncology, Taoyuan General Hospital, Ministry of Health and Welfare, Taoyuan 330, Taiwan; csj995@yahoo.com.tw; 4 Department of Post-Baccalaureate Veterinary Medicine, Asia University, Taichung 413, Taiwan; cch99@asia.edu.tw; 5 Department of Pathology, Hsin-Chu Branch, National Taiwan University Hospital, Hsinchu 300, Taiwan; fox66018@gmail.com; 6 Department of Pathology, Taichung Hospital, Ministry of Health and Welfare, Taichung 403, Taiwan; lukemtlai@gmail.com; 7 Department of Medical Laboratory Science and Biotechnology, Yuanpei University of Medical Technology, Hsinchu 300, Taiwan; tingyu@mail.ypu.edu.tw; 8 Department of Internal Medicine, Hsin-Chu Branch, National Taiwan University Hospital, Hsinchu 300, Taiwan; weishun.yang@gmail.com; 9 Department of Business Administration, Yuanpei University of Medical Technology, Hsinchu 300, Taiwan; chyang@mail.ypu.edu.tw; 10 Department of Biotechnology and Pharmaceutical Technology, Yuanpei University of Medical Technology, Hsinchu 300, Taiwan; 11 Division of Nephrology, Department of Internal Medicine, Taoyuan Branch, Taipei Veterans General Hospital, Taoyuan 330, Taiwan; 12 Department of Nursing, Yuanpei University of Medical Technology, Hsinchu 300, Taiwan

**Keywords:** interleukin-6 receptor, colorectal carcinoma, xenograft, tocilizumab, Ki-67, ERK 1/2, STAT3

## Abstract

The use of tocilizumab against the interleukin-6 receptor (IL-6R) has been demonstrated as inhibiting the progression of diverse cancers in vitro and in vivo. Nonetheless, evidence regarding the anti-tumor effects of tocilizumab on human colorectal carcinoma (CRC) corresponding to IL-6R expression levels remains scarce. To investigate the influence of IL-6R expression, SW480 and HT-29 cells inoculated subcutaneously into NU/NU mice were used as human CRC xenograft models with anti-IL-6R antibody (tocilizumab) therapy. The IL-6R expression levels, histology of CRC growth/invasiveness, and tumor growth-related signaling pathway were estimated by H&E and immunohistochemical staining. SW480 tumor cells with higher IL-6R expression levels showed better responsiveness in tocilizumab therapy than in the treated HT-29 group. Likewise, therapeutic effects of tocilizumab on the proliferative ability with mitotic index and Ki-67 expressions, invasiveness with MMP-9 proteinase expressions, and ERK 1/2 and STAT3 signaling transduction in the SW480 treatment group were superior to the HT-29 treatment group. In light of our results, IL-6R is the key indicator for the efficacy of tocilizumab treatment in CRC xenografts. From the perspective of precision medicine, tumor response to anti-IL-6R antibody therapy could be predicted on the basis of IL-6R expression levels. In this manner, tocilizumab may serve as a targeted and promising anti-CRC therapy.

## 1. Introduction

Interleukin-6 receptor (IL-6R), stimulated by IL-6, triggers intricate cell functions including immune cell activation, hematopoietic stem cell differentiation, cancers, and inflammatory bowel diseases [1,2,3]. Not only our earlier research but also recent studies have pointed out that IL-6 levels in serum or tissue were associated with disease status, including proliferative tumor activity, angiogenesis, metastasis, and patient prognosis in colorectal cancer (CRC) [4,5,6,7,8,9,10]. Indeed, research derived from the Cancer Genome Atlas (TCGA) database proved that the IL-6R/STAT3/miR-34a loop is active in primary human CRCs, contributing to a mesenchymal phenotype of tumor cells, along with invasion and metastasis [11]. In addition to the above research implications, IL-6 signaling transduction could serve as a potential target to treat CRC [12]. Evidence has revealed anti-IL-6 antibodies were responsible for promising strategies to treat various cancers [13]. Notably, our previous CRC cell model demonstrated that anti-IL-6 receptor antibodies suppressed colony formation in soft agar and the invasiveness of SW480, which was stimulated by IL-6 [14]. We subsequently discovered that JAK/STAT3, PI3K/AKT, and MEK/ERK-1/-2 pathways were involved in IL-6-induced clonogenicity and invasiveness of SW480, and the neutralization of IL-6R could reverse the IL-6 effect on signaling transduction, suggesting the potential therapeutic role of the anti-human IL-6R antibodies in CRC cell progression [15]. More recently, we further demonstrated that injection therapy of anti-IL-6R antibodies suppressed the tumor growth of SW480 xenograft and attenuated the proliferation marker Ki-67, along with down-regulating JAK/STAT3, PI3K/AKT, and MEK/ERK-1/-2 signaling pathways [16]. Whether IL-6R expression levels in CRC tumors are capable of affecting the efficacy of anti-IL-6R antibody treatment remains unclear. Based on the different IL-6R mRNA levels in SW480 and HT-29 cells [17], the tumor responses to anti-IL-6R antibody treatment were compared between xenografts of SW480 and HT-29 cells in NU/NU mice, including tumor growth, invasiveness in vivo, and the underlying mechanism. We hypothesized IL-6R expression of CRC could be the key factor to influence the tumor response to anti-IL-6R antibody therapy.

## 2. Results

### 2.1. Human CRC SW480 Tumor Xenografts Expressed Higher Levels of IL-6 Receptor Than HT-29 Xenografts

**Figure 1:**
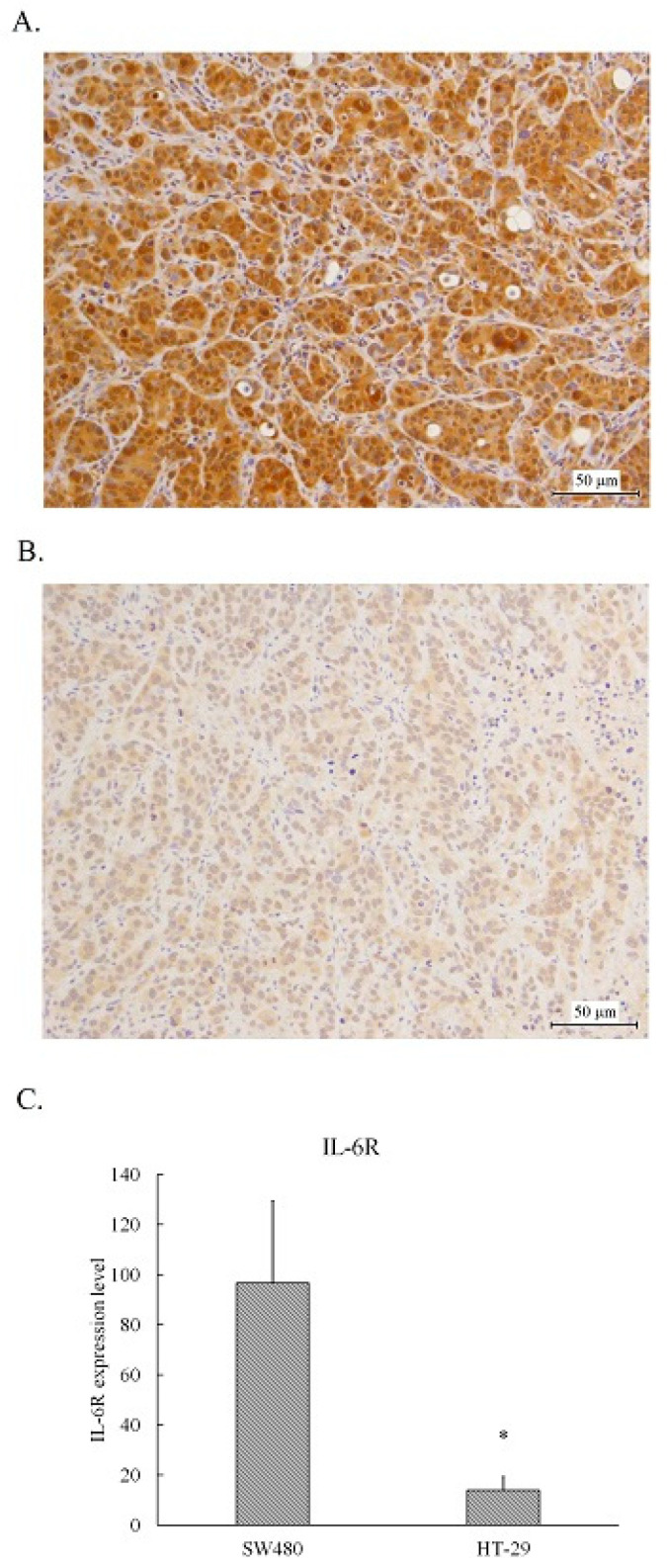
IL-6R expression level in the human colorectal carcinoma SW480 and HT-29 xenografts. Representative images of tumor tissues from the untreated SW480 (**A**) and HT-29 (**B**) were captured. The immunological activity of IL-6R is represented as the average of scores by calculating the percentage and intensity of 10 different areas of a 400X image for each tumor image examined. The statistical results are shown as means ± SEM and significant differences between the SW480 and HT-29 are indicated * *p* < 0.05 (**C**).

The tumors resected from the xenografts inoculated with SW480 or HT-29 cells were fixed and embedded in paraffin, then sliced and stained with IL-6 receptor antibodies using immunohistochemical staining. As shown in Figure 1, medium brown stained cells scattered with some dark brown stained spots were found in SW480 tumors (Figure 1A), whereas light brown stained cells were found in HT-29 tumors (Figure 1B). The IHC overall scores for the of IL-6 receptor in SW480 tumor slices was approximately 100, whereas in the HT-29 tumor slices it was less than 20 (Figure 1C). Obviously, the expression levels of the IL-6 receptor in SW480 tumors was higher than that in HT-29 tumors. 

### 2.2. Inhibitory Effect of Anti-IL-6R Antibody Treatment (Tocilizumab; ACTEMRA^®^) on the Tumor Growth of Human CRC SW480 Xenografts Was Superior to the Treated Group of HT-29 Xenografts

**Figure 2:**
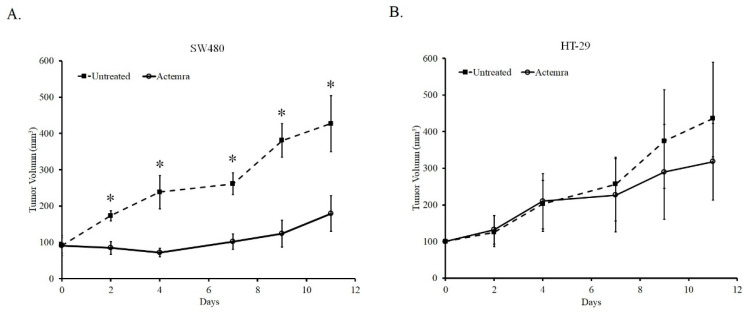
Inhibition effect of anti-IL-6R antibodies on the tumor growth of human colorectal carcinoma SW480 and HT-29 xenografts. SW480 and HT-29 cells (5 × 10^6^) were inoculated subcutaneously into NU/NU mice and peritoneal injection with 1 mg/kg anti-IL-6R antibodies (tocilizumab; ACTEMRA^®^) or PBS were administered three times a week. Tumor volume was measured, and data of each group were averaged from tumor volumes of five mice and are represented as means ± SEM. The tumor growth curve of SW480 (**A**) and HT-29 (**B**) xenografts are shown. * *p* < 0.05.

As shown in Figure 2, the tumor size of both untreated SW480 and HT-29 xenografts gradually grew from a volume of 100 mm^3^ to approximately 400 mm^3^ after 11 days. In the anti-IL-6R antibody (tocilizumab; ACTEMRA^®^, Rhoch Inc., San Francisco, CA, USA) treatment group, the tumor growth was inhibited only in the SW480 xenografts. The tumor was almost sustained at its original size after 7 days and had increased to approximately 160 mm^3^ at the end of the experiment. In contrast to the treated group of SW480, the tumors in the Actemra-treated HT-29 xenograft group showed similar growing potential to the untreated group after 7 days, despite having a slight decrease due to treatment at the end of the experiment. Although the average tumor volume in the treated-HT-29 group was lower than that in the untreated group, the difference between the groups was not statistically significant. The results showed the tumor response to anti-IL-6R antibody therapy was associated with the IL-6R expression levels in the human CRC xenografts. 

### 2.3. Inhibition Effect of Anti-IL-6R Antibody Treatment (Tocilizumab; ACTEMRA^®^) on the Tumor Cell Proliferation of Human CRC SW480 Xenografts Was Superior to the Treated Group of HT-29 Xenografts

**Figure 3:**
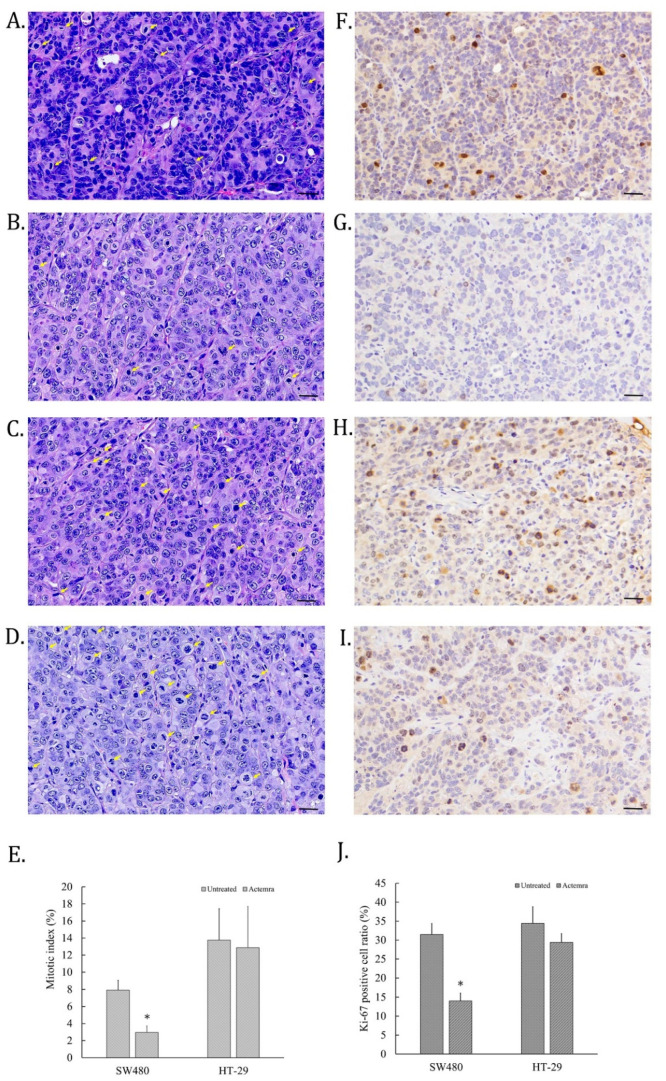
The mitotic index and Ki-67 expression in anti-IL-6R antibody treatment of human colorectal carcinoma SW480 and HT-29 xenografts. At the end of anti-IL-6R antibody treatment, tumors were processed into paraffin slices and then stained with H&E or anti-Ki-67 antibodies for immunohistochemical staining. The images were scanned using a micro scanner, as described in the Section 4. Representative H&E images of tumor tissues from the untreated SW480 (**A**), anti-IL-6R antibody (tocilizumab; ACTEMRA^®^)-treated SW480 (**B**), untreated HT-29 (**C**), and anti-IL-6R antibody-treated HT-29 (**D**) were captured (black bar as 30 μm long). The mitotic cells on the images are indicated by a yellow arrow and the mitotic index of each group is averaged from 10 different areas and is shown as means ± SEM; (**E**). Representative images of Ki-67 immunohistochemical staining of tumor tissue from the untreated SW480 (**F**), anti-IL-6R antibody-treated SW480 (**G**), untreated HT-29 (**H**), and anti-IL-6R antibody-treated HT-29 (**I**) were captured. The Ki-67 positive cells were calculated as a percentage of the total tumor cells in the tumor area of the image, shown as means ± SEM (**J**). * *p* < 0.05.

The cell proliferative ability of CRC in SW480 and HT-29 xenografts was evaluated by H&E staining for mitotic cells and IHC staining for anti-Ki-67 antibodies on the tumor slices. As shown in Figure 3A, some cell nuclei of untreated SW480 tumors disappeared and were replaced with deep dark blue chromosomes, which were identified as the mitotic cells (yellow arrow). However, the mitotic cells in anti-IL-6R antibody-treated SW480 tumors were fewer than in the untreated tumors (Figure 3B). On the other hand, the mitotic cells in the tumors of untreated HT-29 xenografts were more than that of SW480 (Figure 3C), whereas similar numbers of mitotic cells were also found in treated HT-29 tumors (Figure 3D). To further confirm the cell-dividing potential in the tumors of CRC xenografts, Ki-67 expression in the tumor slices was assessed using IHC staining. The Ki-67 positive cells were scattered on the mitotic cells of tumor slices in untreated SW480 and HT-29 cell xenografts (Figure 3E,G). Only a few cells were Ki-67 positive on Actemra-treated SW480 tumor slices (Figure 3F). However, Actemra-treated HT-29 tumors showed more Ki-67 positive cells than the treated SW480 group (Figure 3H). Based on the calculation of the mitotic index on these tumor slices, untreated SW480 showed 8% of total cells, in contrast to only 3% in the Actemra-treated SW480 group (Figure 3I). Both untreated and treated HT-29 showed more than 12% mitotic cells with an insignificant difference between the two groups. The number of dividing cells in each group were further confirmed by Ki-67 positive cells. There was no significant difference between the treated and untreated HT-29 groups. On the contrary, less than 15% Ki-67 positive cells were found in the treated SW480 group, compared with approximately 30% positive cells in the untreated group (Figure 3J). The results indicated that the therapeutic effect of anti-IL-6R on cell proliferative ability was closely correlated with IL-6R expression level in CRC tumors.

### 2.4. Inhibition Effect of Anti-IL-6R Antibody Treatment (Tocilizumab; ACTEMRA^®^) on the Tumor Cell Invasiveness of Human CRC SW480 Xenografts Was Superior to the Treated Group of HT-29 Xenografts

**Figure 4:**
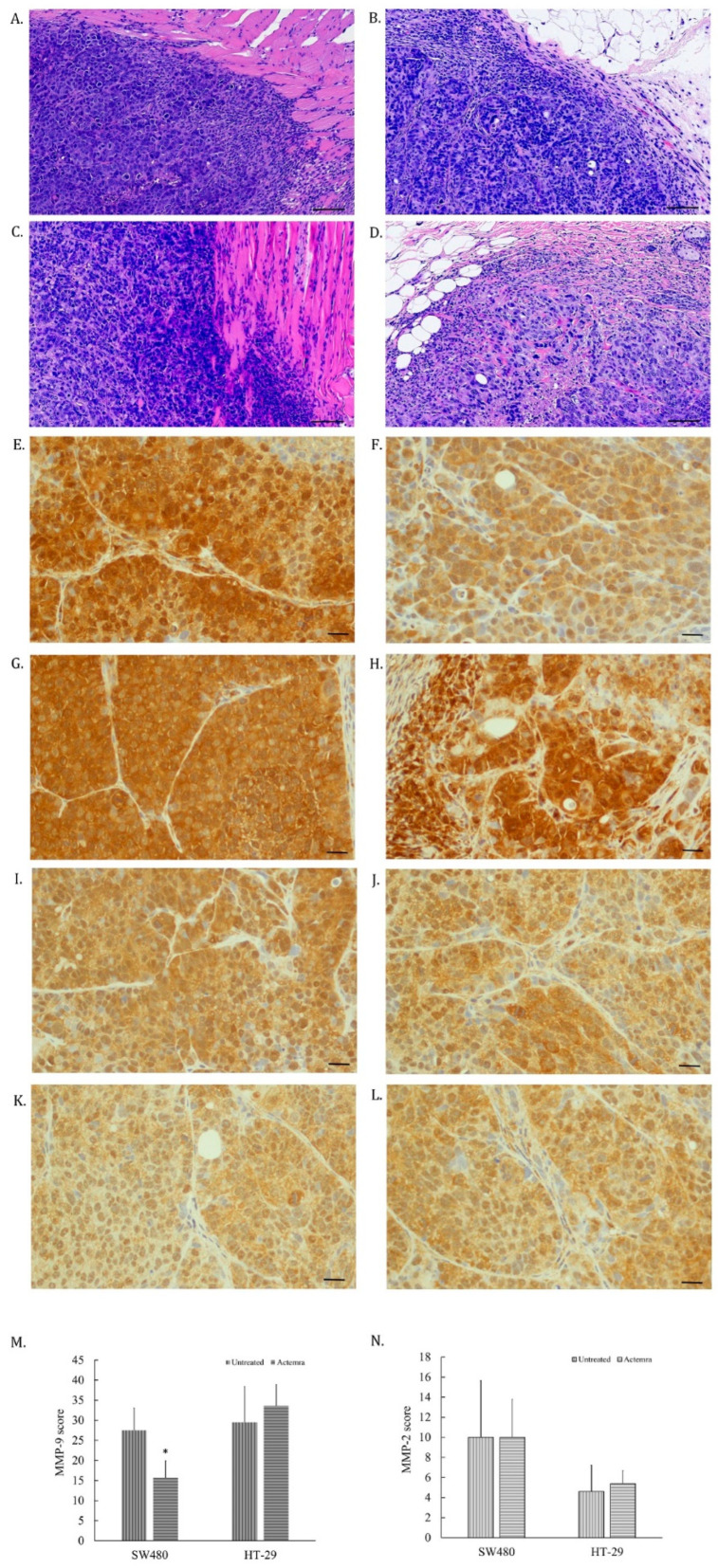
Invasiveness and MMP-9/-2 expression in anti-IL-6R antibody treatment of human colorectal carcinoma in SW480 and HT-29 xenografts. Tumor slices were stained with anti-MMP-9 or anti-MMP-2 antibodies via immunohistochemical staining and the images were scanned using a micro scanner, as described in the Section 4. The tumor invasiveness was first evaluated in the H&E staining slices and representative images showing the edges between tumor and normal tissue were captured (black bar is 60 μm long). The edges of the untreated SW480 tumors had rough, spiculated, and blurry boundaries, and some tumor cells had invaded into the muscle layer of the mouse abdomen (**A**). The edges of the anti-IL-6R antibody-treated SW480 tumors were surrounded by fibrotic tissue with smooth, round, and well-circumscribed boundaries. (**B**). As opposed to Figure 5B, the edges of both untreated (**C**) and tocilizumab (ACTEMRA^®^)-treated HT-29 tumors (**D**) were also rough, spiculated, and blurry, with invasion of infiltrating malignant cells into the muscle layer and fatty tissue of the mouse abdomen. Representative images of MMP-9 staining in tumor slices were captured from the untreated SW480 (**E**), anti-IL-6R antibody-treated SW480 (**F**), untreated HT-29 (**G**), and anti-IL-6R antibody-treated HT-29 (**H**) xenografts (black bar is 30 μm long). MMP-2 images were also captured from the untreated SW480 (**I**), anti-IL-6R antibody-treated SW480 (**J**), untreated HT-29 (**K**), and anti-IL-6R antibody-treated HT-29 (**L**) xenografts. The total score of MMP-9 from each group was averaged from 10 different areas and is shown as means ± SEM (**M**), and MMP-2 shown in (**N**). * *p* < 0.05.

The tumor cells invading the surrounding tissue in SW480 and HT-29 xenografts were evaluated by morphological features using H&E staining and the expression of matrix metaloprotease-2 and -9 (MMP2 and MMP9) on the tumor slices. As shown in Figure 4A, the edge of the tumor was dispersed, and some cancer cells infiltrated into the muscle layer in untreated SW480 tumors. However, anti-IL-6R antibody-treated SW480 tumors were surrounded with fiber tissues (Figure 4B). Both untreated and treated HT-29 tumors showed CRC cells infiltrating into the surrounding tissue (Figure 4C,D). The expression of MMP-9 in untreated SW480 tumor cells (Figure 4E) was higher than in the treated SW480 group (Figure 4F). In contrast, equal expression levels were found in both untreated (Figure 4G) and treated HT-29 tumor cells (Figure 4H). However, the expression of MMP-2 in all tested tumor cells did not seem to differ (Figure 4I–L). The intensity of the MMP-9 expression in the treated SW480 tumors was lower than in the untreated SW480 tumors (Figure 4M), whereas there was no difference between the untreated and treated HT-29 tumors (Figure 4N). The results revealed that the invasion potential of SW480 tumors was suppressed by anti-IL-6R antibody treatment through suppressing MMP-9 expression in treated tumors, corresponding to the expression level of the IL-6R in the tumors.

### 2.5. Inhibition Effect of Anti-IL-6R Antibody Treatment (Tocilizumab; ACTEMRA^®^) on Erk-1/-2 and STAT3 Signaling Transduction of Human CRC SW480 Xenografts Was Superior to the Treated Group of HT-29 Xenografts

**Figure 5:**
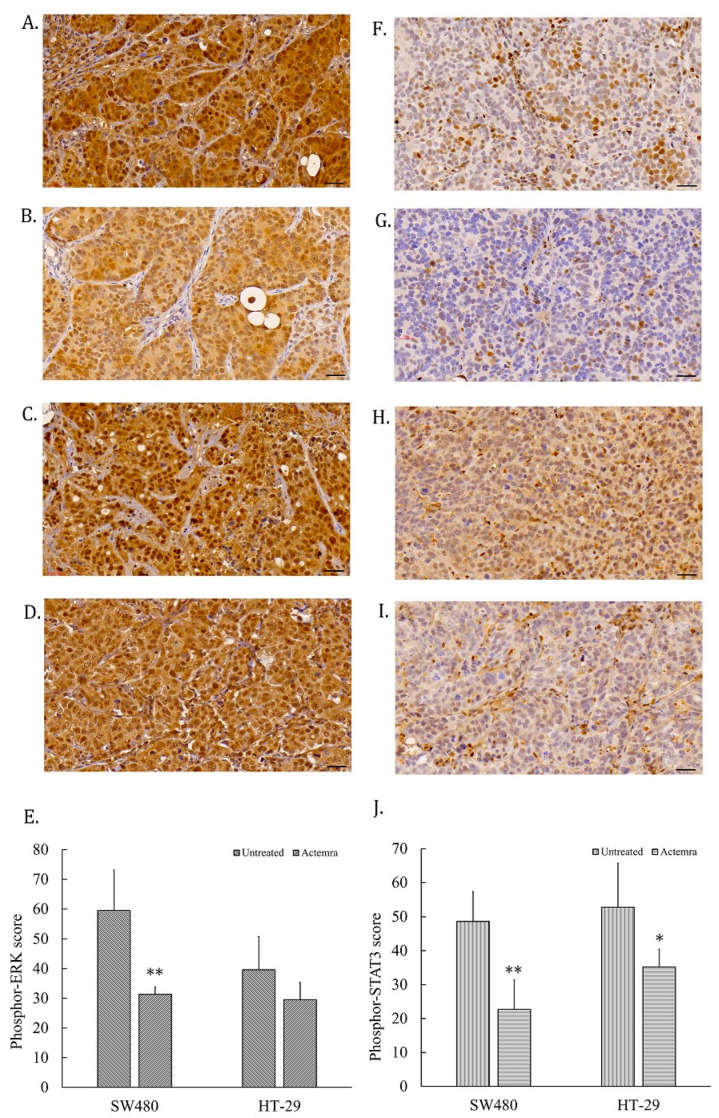
Signaling transduction in anti-IL-6R antibody treatment of human colorectal carcinoma SW480 and HT-29 xenografts. Tumor slices were stained with anti-phosphor-Erk-1/-2 and phosphor-STAT3 antibodies for immunohistochemical staining, and the images were scanned using a micro scanner, as described in the Section 4. Representative images of phosphor-Erk-1/-2 staining in tumor slices were captured from the untreated SW480 (**A**), anti-IL-6R antibody (tocilizumab; ACTEMRA^®^)-treated SW480 (**B**), untreated HT-29 (**C**), and Actemra-treated HT-29 (**D**) xenografts (black bar is 30 μm long). The total scores of phosphor-Erk-1/-2 from each group are averaged from 10 different areas and shown as means ± SEM (**E**). Phosphor-STAT3 images were also captured from the untreated SW480 (**F**), Actemra-treated SW480 (**G**), untreated HT-29 (**H**), and Actemra-treated HT-29 (**I**) xenografts. The total scores of phosphor-STAT3 from each group were averaged from 10 different areas and are shown as means ± SEM (**J**). * *p* < 0.05 and ** *p* < 0.01.

The tumor slices from human CRC SW480 and HT-29 xenografts were subjected to assessment of the IL-6R signaling pathways, including phosphor-STAT3 and phosphor-Erk-1/-2 levels in the untreated and treated groups. As shown in Figure 5A, the phosphor-Erk-1/-2 staining intensity was a strong positive (3+) in almost all the tumor cells from the untreated SW480 group, whereas the intensity was only 1 to 2 plusses within the treated SW480 tumor cells (Figure 5B). The tumor cells showed the intensity of 2 to 3 plusses from the untreated HT-29 group (Figure 5C), and 2 plusses from the treated HT-29 group (Figure 5D). The total intensity score of phosphor-Erk in the untreated SW480 tumor cells was approximately 60 via a semi-quantitative analysis, compared with 30 in the anti-IL-6R antibody-treated SW480 tumors. The phosphor-Erk staining intensity differed significantly between the untreated and treated groups (*p* < 0.01). On the other hand, the score was approximately 40 in untreated HT-29 tumors and 30 in treated HT-29 tumors, but there was no significant difference between these two groups (Figure 5E). Phosphor-STAT3 staining was a strong positive in approximately half of the tumor cells from the untreated SW480 tumors (Figure 5F) and HT-29 (Figure 5H), and decreased in the treated groups of SW480 (Figure 5G) and HT-29 (Figure 5I). The phosphor-STAT3 staining of untreated SW480 tumor cells scored approximately 50 via a semi-quantitative analysis, which was significantly different from the treated group, with a score of 23 (*p* < 0.01) (Figure 5E). The phosphor-STAT3 staining of untreated HT-29 tumor cells scored approximately 60, compared with 38 in the treated HT-29 group (*p* < 0.05) (Figure 5J). The phosphor-STAT3 staining intensity differed significantly between the two groups.

## 3. Discussion

Emerging evidence has illustrated that the IL-6/IL-6R gp130 inflammation signaling pathway plays a crucial role in the promotion of carcinogenesis in CRC [18,19,20]. Targeted anti-IL-6R antibody therapy for CRC was capable of providing an adjuvant strategy [21,22]. Our previous studies showed that anti-IL-6R antibodies potentially inhibited tumor growth and invasiveness in vitro and in vivo through interfering with IL-6 signaling transduction in IL-6R expressing CRC cell lines, including Erk-1/-2, STAT3 and AKT phosphorylation/activation [15,16]. Theoretically, the tumor response to targeted therapy is closely associated with expression levels of target proteins in/on cancer cells [23]. In the present study, we attempted to further investigate whether IL-6R expression level is the important predictor for the tumor response of CRC to anti-IL-6R antibody treatment. Two CRC cell lines, SW480 and HT-29, were chosen after evaluating the IL-6R mRNA levels in an earlier study that showed SW480 cells exerted higher IL-6R levels than HT-29 cells [17]. The tumor slices from the xenografts of these two cell lines showed different protein levels of IL-6R. Indeed, the SW480 tumors exerted a more potent IL-6R intensity than HT-29. Also, the inhibitory effect of anti-IL-6R antibodies on tumor growth in SW480 xenografts was more powerful than in HT-29. The tumor growth with anti-IL-6R antibody therapy further represented attenuated expressions of the mitotic index and Ki-67 staining. Specifically, SW480 tumors posed high levels of IL-6R that anti-IL-6R antibody treatment effectively suppressed in the mitotic cells. In contrast, the HT-29 tumors with low IL-6R expressions represented equal results of mitotic index and Ki-67 staining between the untreated and anti-IL-6R antibody-treated groups. The results indicate that CRC tumor cells with higher IL-6R expression are more sensitive to anti-IL-6R antibody treatment.

The two human CRC cell xenografts represented different invasiveness abilities corresponding to IL-6R expression levels. The tocilizumab-treated SW480 tumors were surrounded by fibrotic tissue that formed a barrier-like structure to avoid CRC cell infiltration, as described in our previous report [16]. However, the tumor edge of the same treatment of HT-29 xenografts seemed to be unclear, and was similar to the untreated SW480 or HT-29 tumors. Meanwhile, there were several cancer cells infiltrating into surrounding muscle or fatty tissue. Several studies, along with our research, revealed that the expression of invasiveness-related proteinases such as MMP-9/MMP-2 were inhibited by interfering with the IL-6 signaling transduction that suppressed CRC cell invasiveness in vitro [15,24,25,26]. Herein, we found the expression of MMP-9 was suppressed by tocilizumab treatment in human CRC SW480 xenografts. Nonetheless, the MMP-9 level did not significantly differ between the untreated and treated HT-29 groups. The IL-6R expression level in CRC cells would be the key factor for the effect of tocilizumab on the expression of invasiveness-related proteinases. The MMP-2 expression in all groups was not changed, suggesting the underlying mechanism of CRC tumor invasiveness is extremely intricate. Previous reports revealed that cell signals triggering ERK phosphorylation/activation serve as an important regulatory mechanism to modulate MMP-9/MMP-2 expressions [15,27,28]. In the current study, phosphor-ERK was not suppressed by tocilizumab in HT-29 tumors. Our prior study demonstrated that only simultaneously suppressing ERK and AKT phosphorylation was capable of inhibiting the expression of MMP-9/MMP -2 and the invasiveness of CRC cells, providing the possible reason that explains the discrepancy between invasiveness-related experiments [15]. Last but not least, tocilizumab suppressed the phosphor-STAT3 signaling in both the treated SW480 and HT-29 groups, indicating that JAK/STAT-3 signaling transduction is more sensitive to IL-6/IL-6R signaling blockade, even in the lower IL-6R-expressing HT-29 cells.

Some limitations are recognized in our study. Although we explained that the differences observed in this study are attributable to the different expressions of IL-6R, the model used is not robust enough. In fact, there are many other genetic differences with different IL6R mRNA expressions between these two cell lines [29]. As for the Ras/RAF/MEK/MAPK sequence related to IL6/IL6R signaling, the mutations of SW480 and HT29 are Ras and RAF, respectively. Because IL6R has a STAT3 signaling pathway, disrupting the upstream signal IL6 cannot prevent cell growth. Our previous articles confirmed that the IL6R antibody tocilizumab can bypass Ras mutation and directly affect downstream MAPK in SW480, thereby slowing down the growth of transplanted tumors [15,16]. This research further explores whether the expression level of IL6R will affect the inhibitory effect of tocilizumab on CRC xenografts. The current results confirm that the discrepancy in mRNA expression exists not only in cell models but also in xenograft models. Furthermore, because the inhibitory effect of tocilizumab bypasses Ras and directly affects downstream MAPK, we infer that the inhibitory effect on HT29 with RAF mutation may be worse than SW480. The main phenomenon results from the synergism of low IL6R expression and the influence of RAF mutation. Our previous studies revealed that the inhibitory effect on cell invasion was related to the inhibition of MMP9 [15,16]; however, we further confirm the therapeutic effect on the CRC invasion into surrounding tissues through in vivo model.

## 4. Materials and Methods

### 4.1. Materials

Primary antibodies including anti-ERK-1/-2, anti-phospho-ERK-1/-2 (The202/Tyr204), anti-STAT3, anti-phosphor-STAT3 (S727), anti-AKT, anti-phospho-AKT, anti-Ki-67, and secondary antibody conjugated with poly-peroxidase and DAB chromogen for immunohistochemical (IHC) staining were purchased from Abcam (Cambridge, MA, USA) and GeneTex (Hsinchu, Taiwan).

### 4.2. Cell Culture

Cell culture medium and other supplements were purchased from Gibco Ltd. (Paisley, UK). CRC cell line SW480 and HT-29 were purchased from the Bioresource Collection and Research Center, Taiwan, and cultured in 90% medium supplemented with 10% heat-inactivated FBS, 25 U/mL penicillin, and 25 μg/mL streptomycin at 37 °C and a water-saturated atmosphere. All experiments were carried out on cell lines passaged 5–20 times.

### 4.3. Animal Study

Animal experiments were performed according to the Guide for the Care and Use of Laboratory Animals (8th Edition), and the detailed protocol used here was reviewed and approved by the Institutional Animal Care and Use Committee (IACUC No. 2017-234) of the China Medical University (Taichung, Taiwan). The animal study was described as per our previous report [16]. Briefly, male nude mice (6-week-old) (strain NU/NU; BioLASCO Taiwan Co., Ltd., Taipei, Taiwan) cultivated under pathogen-free conditions were inoculated with 100 μL cell suspension of 5 × 10^7^ cells/mL SW480 or HT-29 cells using a 1 mL syringe and a 27-gauge needle. After one week, the 7-week-old mice with approximately 100 mm^3^ of the tumor size were divided into four groups. Each group contained 5 animals. The treated groups underwent peritoneal injection with 1.0 mg/kg anti-IL-6R antibodies (tocilizumab; Rhoch Inc., San Francisco, CA, USA) in 0.1 mL PBS on days 2, 4, 7, 9, and 11. The untreated groups were injected with an equal volume of PBS as a replacement for the anti-IL-6R antibodies. We used a sensitive electronic scale to weigh an empty scooping container as the first measurement. Then, the mouse placed inside the container was weighed as the second measurement. The difference in weight between the two measurements was recorded as the weight of the mouse. Through the above method, the mice were weighed before drug administration and sacrifice. The length, width, and height of the tumors were measured with vernier calipers on days 0, 2, 4, 7, 9, and 11, and the volume was calculated (V = length × width × height/2). The mice were sacrificed when 9 weeks old, and the tumors along with other organs, such as the liver, lungs, spleen, heart, and kidneys, were removed surgically and fixed in 3% formalin/PBS.

### 4.4. Tissue Processing and H&E Staining

The collected tumors or other visceral tissues were first washed with PBS to remove external blood, and then soaked in 3% formaldehyde-PBS (neutral formalin) at room-temperature overnight. The fixation time did not exceed 24 h. The details of the procedures were essentially in accordance with the previous study [16]. Briefly, the formalin-fixed tissues in plastic tissue support racks were dehydrated via sequential immersion in 50%, 70%, 85%, 95%, and 100% ethanol, and then xylene under an automatic dehydrator machine, and finally embedded in the molten paraffin wax. After slicing the paraffin-embedded tissues with a microtome (Leica RM2125 RTS; Leica Microsystems Inc., Seattle, WA, USA), the 2–3-μm-thick tissue sections were fixed on glass slides by heating at 60 °C overnight. For hematoxylin and eosin (H&E) staining, the 65 °C heat de-waxed paraffin-embedded tumor slices were washed in xylene and rehydrated with a series of alcohol gradients (absolute alcohol, 95% alcohol, and 80% alcohol). The rehydrated tissue sections were stained with Mayer’s H&E protocol and then underwent the dehydration process as described in our previous report [16]. The mounted slices were scanned by a digital pathology scanner (MoticEasyScan; Motic Inc., Xiamen, China) to obtain the digital microscopy images on a computer using the image program (DSAssistant; Motic Inc., Xiamen, China).

### 4.5. Immunohistochemical (IHC) Staining

The details of the procedures were essentially in accordance with our previous study [16]. Briefly, the tumor slices were rehydrated with a series of alcohol gradients (absolute alcohol, 95% alcohol, and 80% alcohol) and finally, immersed in TBS (Tris-buffered saline). After antigen retrieval in 95 °C heated citrate buffer and de-activated endogenous peroxidases with 3% H_2_O_2_, tissue sections in the slices were incubated with anti-Ki-67, ERK-1/-2, phosphorylated ERK-1/-2, STAT3, phosphorylated STAT3, matrix metalloproteinase (MMP)-9, and MMP-2 antibodies at the indicated dilution ratios. The location and intensity of each antigen on the tissue sections were visualized using the LSAB staining system and hematoxylin as the counterstain. The stained slices were dehydrated and scanned as high-resolution digital images using a digital pathology scanner. The overall staining intensity was typically classified into four categories: negative (0), weak (1 plus), moderate (2 plusses), and strong (3 plusses). The immunological activities of each antigen with the intensity scores were calculated by semi-quantitative analysis, as described in prior studies [16,30].

### 4.6. Statistical Analysis

The data were analyzed using the Kruskal–Wallis test, which is a nonparametric test, to evaluate significant differences between the control group and the treatment groups. If significant differences existed, the multiple comparison test was then used for identification. Differences among group means were considered significant at *p* < 0.05.

## 5. Conclusions

Tocilizumab is a humanized monoclonal antibody used directly against IL-6R. The human CRC SW480 xenografts with higher IL-6R expression levels showed better responsiveness than the treated HT-29 group. Meanwhile, such therapeutic effects on proliferative ability with mitotic index and Ki-67 expressions, invasiveness with MMP-9 proteinase expressions, and ERK 1/2 and STAT3 signaling transduction in the SW480 treatment group were superior in the HT-29 treatment group. In light of our results, IL-6R is the key indicator for the efficacy of tocilizumab treatment in CRC xenograft models. From the perspective of precision medicine, tumor response to anti-IL-6R antibody therapy could be predicted on the basis of IL-6R expression levels. In this manner, tocilizumab could serve as a targeted and promising anti-CRC therapy.

## Data Availability

All data used to support the findings of this study are available from the corresponding author, Chang, J.-F., upon reasonable request.
